# Spinal Caries

**Published:** 1894-12

**Authors:** 


*Spinal Caries.* By NOBLE SMITH. Pp. vi., 146. London: SMITH, ELDER & Co. 1894.

This is a practical treatise, giving the results of the author's very considerable experience on the subject of spinal curvature. It is illustrated with numerous drawings made by the author himself, most of which are of value in explaining the text, although they may not be altogether artistic.

Mr. Noble Smith is very emphatic in condemning Sayre's plaster-of-Paris jackets. He records numerous instances where pressure sores have been caused by improper application or imperfect attention afterwards; but he completely ignores the hundreds and thousands of cases which have been cured among hospital patients by the proper use of this invaluable mode of treatment.

The only kind of apparatus which meets with the approval of the author is what he calls the "adaptable metal splint," consisting of an abdominal belt, with two padded plates for the sides of the angle of disease and shoulder straps, the whole being held together by two upright steel bars. We do not doubt the efficacy of this apparatus when carefully fitted and applied, but we altogether deny that it ought to replace entirely the use of poro-plastic felt or plaster-of-Paris jackets.

In the treatment of spinal abscesses, the author has apparently met with such extraordinary success that there can be nothing left to be desired. For fourteen years he has treated all chronic abscesses by opening freely, and afterwards syringing them out daily with carbolic acid 1 in 40. With such treatment he has never known any abscess to become contaminated, and has never produced anything but beneficial results (p. 113). Would that we could all obtain similar results!

